# Toward Implementation of Mosquito Sterile Insect Technique: The Effect of Storage Conditions on Survival of Male *Aedes aegypti* Mosquitoes (Diptera: Culicidae) During Transport

**DOI:** 10.1093/jisesa/iey103

**Published:** 2018-11-07

**Authors:** Hae-Na Chung, Stacy D Rodriguez, Kristina K Gonzales, Julia Vulcan, Joel J Cordova, Soumi Mitra, Christopher G Adams, Nathan Moses-Gonzales, Nicole Tam, Joshua W Cluck, Geoffrey M Attardo, Immo A Hansen

**Affiliations:** 1 Department of Biology, New Mexico State University, Las Cruces, NM; 2 Institute of Applied Biosciences, New Mexico State University, Las Cruces, NM; 3 Department of Entomology, Michigan State University, East Lansing, MI; 4 M3 Consulting Group, Unmanned Aircraft Systems Initiatives, Dayton, OH; 5 Department of Entomology and Nematology, University of California, Davis, Davis, CA

**Keywords:** *Aedes aegypti*, sterile insect technique, shipping, temperature, compaction

## Abstract

Sterile insect technique (SIT) is a promising, environmentally friendly alternative to the use of pesticides for insect pest control. However, implementing SIT with *Aedes aegypti* (Linnaeus) mosquitoes presents unique challenges. For example, during transport from the rearing facility to the release site and during the actual release in the field, damage to male mosquitoes should be minimized to preserve their reproductive competitiveness. The short flight range of male *Ae. aegypti* requires elaborate release strategies such as release via Unmanned Aircraft Systems, more commonly referred to as drones. Two key parameters during transport and release are storage temperature and compaction rate. We performed a set of laboratory experiments to identify the optimal temperatures and compaction rates for storage and transport of male *Ae. aegypti*. We then conducted shipping experiments to test our laboratory-derived results in a ‘real-life’ setting. The laboratory results indicate that male *Ae. aegypti* can survive at a broad range of storage temperatures ranging from 7 to 28°C, but storage time should not exceed 24 h. Male survival was high at all compaction rates we tested with a low at 40 males/cm^3^. Interestingly, results from our ‘real-life’ shipping experiment showed that high compaction rates were beneficial to survival. This study advances key understudied aspects of the practicalities of moving lab-reared insects into the field and lies the foundation for further studies on the effect of transport conditions on male reproductive fitness.

The yellow fever mosquito, *Aedes aegypti* (Linnaeus), is a highly anthropocentric disease vector species with worldwide distribution (Powell and Tabachnick 2013). It is the principal vector of some important arboviruses, including yellow fever, dengue, chikungunya, and zika (Mayer et al. 2017). Originally from Africa, this pestiferous insect is now found in tropical, subtropical, and temperate regions throughout the world. The spread of these mosquitoes has brought the threat of these viral diseases to new geographic areas with further spread predicted as a result of temperature changes from global warming. Conventional vector control activities for this species include habitat reduction and the use of larvicides and adulticides (Maciel-de-Freitas et al. 2014). Rapid evolution of insecticide resistance in *Ae. aegypti* populations has become a major problem that justifies the development of novel control strategies for this vector (Deming et al. 2016).

Sterile insect technique (SIT) is a targeted, environmentally friendly tactic for insect control where large quantities of sterile males are released into targeted areas, where they compete with the wild male population for mating opportunities with females (Dyck et al. 2006, Thome et al. 2010). Matings between wild females and sterilized males do not produce viable eggs. Over time this technique reduces the number of individuals in the local population, which leads to a decline in and eventually a crash of the entire population (Benedict and Robinson 2003, Klassen 2009).

The concept of SIT to control insect pest populations was developed in the early 1900s; however, it was not implemented until the 1950s (Boyer 2012). The technique was first applied for the control of the screwworm fly by Bushland and Knipling (Bushland et al. 1955, Knipling et al. 1968, Bouyer and Lefrancois 2014). There are a number of examples of SIT programs that have successfully eradicated their target pests. Three such examples are as follows: 1) the North and Central American screwworm fly (*Cochliomyia hominivorax* Coquerel [Diptera: Calliphoridae]), which was eradicated from southern United States, Mexico, and all of Central America (Atzeni et al. 1994, Krafsur and Lindquist 1996, Wyss 2000); 2) the Mediterranean fruit fly (*Ceratitis capitata* Wiedemann Wiedemann [Diptera: Tephritidae]), eradicated from Central America and Mexico (Hendrichs et al. 2002, Juan-Blasco et al. 2014); and 3) the tsetse fly (*Glossina austeni* Newstead [Diptera: Glossinidae]), which was eradicated from the island of Zanzibar in Tanzania (Vreysen et al. 2000, Dyck et al. 2006, Abd-Alla et al. 2013).

Several critical components are required for a SIT program to be successful. The rearing program must be able to raise large numbers of healthy insects that exhibit normal behaviors. Sterilization, or inherited sterility, must be highly efficient with little or no negative impact on the fitness of the males so they can compete successfully for wild females (Rodriguez et al. 2013). Insects must be packaged, chilled, held for a time, and transported to the field without killing or damaging the insects (Tsitsipis 1977, Lux et al. 2002). Releases must be timed correctly and targeted to areas where wild populations are found, so that sterile males have a high probability of mating with wild females within their life span. In addition, the frequency of releases and the quantity of insects per release must be optimized (White et al. 2010).

The implementation of a SIT program for mosquitoes in general, and *Ae. aegypti* in particular, poses several unique challenges. A key challenge with *Ae. aegypti* is their short flight range. The average flight range of *Ae. aegypti*, regardless of sex, is reported to be only ca. 25–200 m (Harrington et al. 2005). By comparison, the screwworm fly has a flight range of 10–20 km in warm, humid environments, to as much as 300 km in arid climates (Parish 1937, Baumhover et al. 1955). The limited flight range of *Ae. aegypti* requires us to consider different release strategies to effectively cover a given area when administering a mosquito SIT program with this species. Mosquito releases using Unmanned Aircraft Systems (UAS), also known as drones, carrying a mosquito storage and release system can provide a solution to this problem. By utilizing UAS, the distribution area can be increased compared with ground release strategies (Liew and Curtis 2004). In the past few years, several attempts have been made to implement the release of sterile male mosquitoes (among other insect species) with remote-controlled drones (Tan and Tan 2013, Mubarqui et al. 2014, Maria de Genaro Chiroli et al. 2016). Recently, chilled adult release has been proposed for the aerial release of sterile insects (Tan and Tan 2013) and integrated in a SIT release device (Mubarqui et al. 2014).

Important practical issues for drone release of sterile male mosquitoes are the temperature and compaction conditions during their transport from the rearing/sterilization site to the release site. Insects held at reduced temperatures are less likely to damage themselves and other contained insects if their metabolism is slowed temporarily. Changes in temperature and humidity have been shown to affect flight performance and survival of female *Ae. aegypti* (Rowley and Graham 1968, Costa et al. 2010). To increase economy and effectiveness of releases, the maximum number of male mosquitoes per release containers—that does not impact fitness—must be derived. If the insects are too densely compacted and/or held at unsuitable temperatures, they are likely to be damaged or killed before release. On the other hand, providing males with too much space or holding them at too warm a temperature might lead to increased activity inside the containers, resulting in damaged legs and wing structures and an increase in mortality. In a recent study by Culbert et al. (2017) on transport conditions for *Anopheles arabiensis* mosquitoes the authors found that temperatures between 4 and 10°C as well as compaction did not have any significant effects on survival.

In this article, we explore optimal holding temperatures and compaction rates for *Ae. aegypti* males to gain information for future transport, storage, and drone-release protocols.

## Materials and Methods

### Mosquito Culture

The *Ae. aegypti* ROCK strain was obtained from MR4, the Malaria Research and Reference Reagent Resource Center (Adams et al. 2000). The protocol for rearing is described in Marquardt et al. (2004). Approximately 2,000–5,000 mosquitoes were reared for each experimental replicate. Larvae were reared in 40.64 × 50.8 cm plastic developing trays filled with approximately 2.5 liters of deionized water. Pans were housed in an insect chamber at 28°C and 80% humidity and set for a photoperiod of 14:10 (L:D) h. The larvae were fed Special Kitty Cat food pellets (The J.M. Smucker Co., Orrville, OH) ad libitum. Pupae were collected daily and transferred to BugDorm-1 Insect Rearing Cages (30 × 30 × 30 cm, BugDorm Store, Taichung, Taiwan). Adults were provided with 20% sucrose solution in 25-ml Eppendorf flasks with a cotton wick. Males were collected in aspirator tubes using a battery-powered aspirator (BioQuip, Rancho Dominguez, CA).

### Temperature Effects on Mosquito Survival Rates Over Time

Males were anesthetized by placing the aspirator tubes on ice for approximately 10 min and transferred to the surface of a petri dish that was placed on ice. Males were separated from females using pointed feathers and tweezers, counted, and placed into 50-ml Falcon tubes (USA Scientific, Ocala, FL). Holes to allow for airflow were drilled into the tube caps and in the bottom of the tubes using 16-gauge injection needles (MedLab supplies, Pompano Beach, FL). Tubes were placed in an Incufridge incubator (Revolutionary Science, Shafer, MN) set to 80% humidity and at 7, 14, 21, or 28°C. For these experiments, a replicate consisted of a tube containing 20 male mosquitoes with *n* = 5 for each temperature. Mortality was recorded every 24 h for 96 h, or until all males were dead. After 96 h, mosquitoes were taken out of the tubes and transferred into BugDorm Insect Rearing Cages (12 × 12 × 12 cm, BugDorm Store, Taichung, Taiwan). Water with 20% sucrose was offered in 25-ml Eppendorf flasks with a cotton wick, and the males were left to recover for no longer than 2 h. Mosquitoes that were unable to fly were counted as dead.

### Survival by Compaction and Temperature

Compaction chambers with a volume of 10 cm^3^ were constructed from 50-ml Falcon tubes by cutting them with a hand saw at the 40-ml line. The cut end of the Falcon tubes was sealed off using a fine mesh attached with a hot glue gun (Add Tech, Hampton, NH). Holes were drilled into the lids using 16-gauge needles to allow for air flow. Males were anesthetized by chilling on ice (as described earlier), counted, and placed into the compaction chambers. The compaction chambers were then placed in an incubator set to 80% humidity and at 7, 14, 21, and 28°C, and held for 24 h. At each temperature, four levels of compaction were created by adding 100, 200, 400 and 800 males per compaction chamber. After 24 h, the mosquitoes were transferred to BugDorm cages (12 × 12 × 12 cm, BugDorm Store, Taichung, Taiwan) provisioned with sugar water as described earlier and allowed to recover for 2 h. Mosquitoes unable to fly after 2 h were counted as dead. For each temperature, four replicates were performed.

### Mosquito Survival Rates With Compaction at 14°C

A second compaction study was performed using 5-ml syringes. Male mosquitoes were compacted at the same volume (1 cm^3^) at numbers of 10, 20, 30, 40, 50, 60, 70, 80, 160, and 240 male mosquitoes. Supp File 1 (online only) shows the procedure of compacting 240 males in a syringe. Males were temporarily anesthetized on ice, counted individually into syringes (which were blocked off at the application end using cotton ball material), and transferred to an incubator at 14°C and 80% humidity. After 24 h, the mosquitoes were transferred to BugDorm cages with sugar water and allowed to recover for 2 h. Mosquitoes that could fly were counted toward survival numbers, and mosquitoes unable to fly were considered dead and counted toward mortality numbers.

### Shipping Assay

Ten-milliliter syringes were prepared by drilling eight holes next to the nozzle of the syringe using 16-gauge injection needles. The syringes were washed briefly with soap water, rinsed with distilled water, and air dried. The needle hub was closed off with a tip cap that was perforated with a 16 gauge injection needle. Various numbers of male mosquitoes (10, 40, 240) were compacted in the 10-ml syringes to a volume of 1 cm^3^ by pushing the plunger slowly to the 1-ml mark (see Supp File 1 [online only]). The syringes were put into a Styrofoam container with a cooling element at 4°C and an RC51 temperature logger (Elitech, Milpitas, CA). The Styrofoam box was shipped via airfreight overnight courier service from Las Cruces, NM, to Davis, CA. On arrival, the males were immediately released from the syringes onto wet paper towels inside Bugdorm mosquito cages. After 2 h, survival rates were determined, and the survivors were checked for damage using a Laxco MZS33 Series Stereo Microscope (Fisher Scientific, Ann Arbor, MI).

### Statistical Methods

Kaplan–Meier survival analysis tests were conducted using XLSTAT (Addinsoft, New York, NY) to determine the statistical significance in differences in survival of male mosquitoes under different temperatures and compaction rates. A Mann–Whitney analysis was used to determine the statistical significance for temperature and cooling and compaction assays (INSTAT, GraphPad Software, La Jolla, CA).

## Results

### Storage Temperature Affects Mosquito Survival During Incubator Storage

**Fig. 1.**
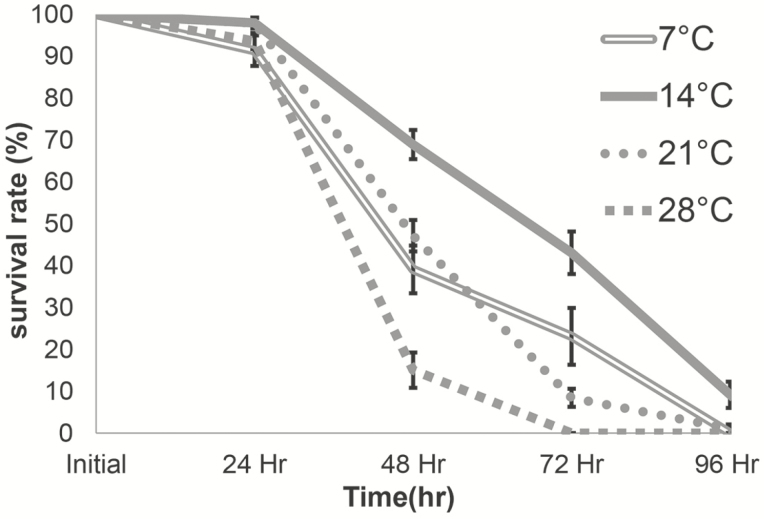
Temperature effects on mosquito survival rates over time. Mean survival rates (± SEM) at 24, 48, 72, and 96 h when mosquitoes were held at 7, 14, 21, and 28°C. Data are representative of at least three independent trials. The Kaplan–Meier survival analysis was performed to determine statistical differences between the curves.

We started this study by determining suitable storage temperatures for male mosquitoes that were stored in 10 cm^3^ containers in groups of 20 males. Fig. 1 shows the survival curves of these male mosquitoes at different storage temperatures. We observed high survival rates for all temperature regimens at the 24-h time point. Mosquitoes held at 14°C had a significantly higher percent survival at the 48- and 72-h time points than all the other temperature regimens. There was no statistically significant difference in survival between the mosquitoes held at 7 and 21°C at any time point, whereas mosquitoes held at 28°C had the significantly lower survival rates than the other temperature groups.

### Compaction Affects Mosquito Survival During Incubator Storage

**Fig. 2.**
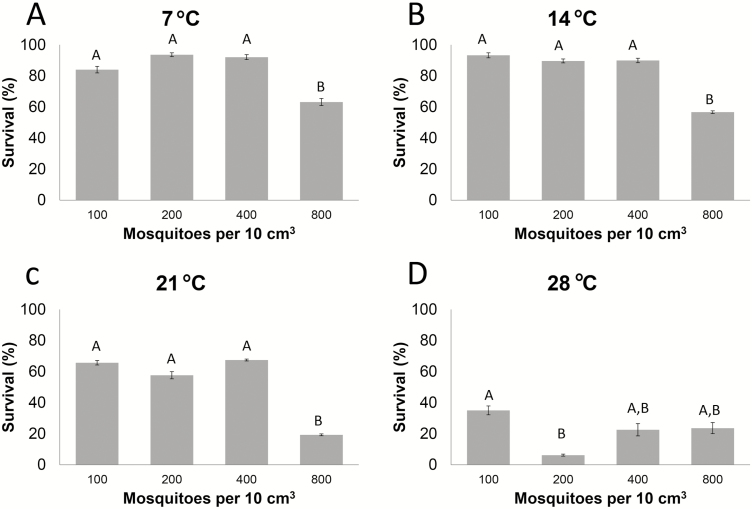
Survival by compaction and temperature. Mean survival rates (± SEM) after 24 h of storage at four different temperatures and four different levels of compaction. Data are representative of at least three independent trials. The Mann–Whitney test was performed to determine statistical differences between the means. Different letters indicate significant difference at *P* < 0.05.

We tested male mosquito survival at varying temperatures (7, 14, 21, and 28°C) and different compaction numbers (100, 200, 400, and 800) in the above-described setup. When observing mosquito survival at 7°C, there was no significant difference between the different compaction rates (100, 200, and 400 mosquitoes/10 cm^3^); however, there was a statistically significant decrease in survival at 800 mosquitoes/10 cm^3^ (Fig. 2A). At 14 and 21°C storage temperatures, 800 males/10 cm^3^ was the only compaction rate that resulted in a survival value that was statistically lower than the other survival values (Fig. 2B and C). Storage at 28°C resulted in low survival rates at all compactions tested (Fig. 2D).

### Cooling and Compaction Affect Mosquito Survival in Syringe–Incubator Storage Assays

**Fig. 3.**
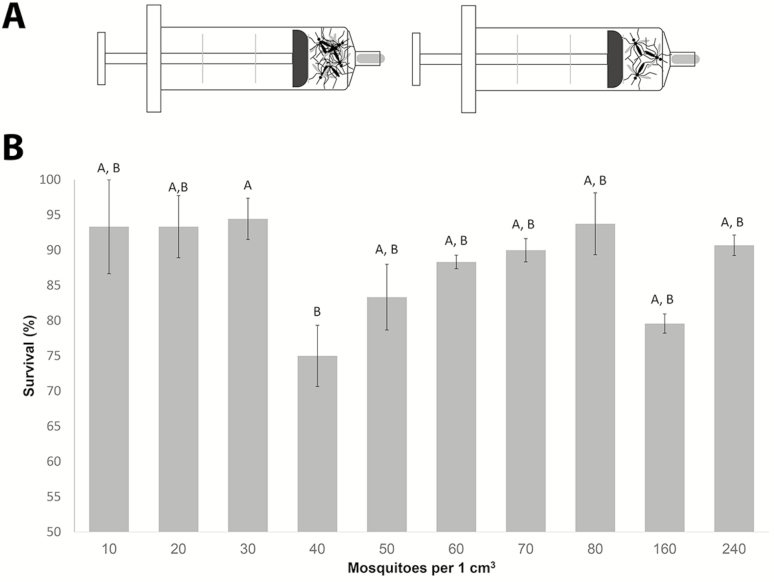
Mosquito survival rates after 24-h compaction at 14°C. (A) Experimental setup: A designated number of mosquitoes were compacted to 1 cm^3^ space of a 10-ml syringe and stored at 14**°**C for 24 h in an incubator. (B) Mean survival rates (± SEM) of male mosquitoes at the end of the storage time. Data are representative of at least three independent trials. The Mann–Whitney test was performed to determine statistical differences in comparison to whole blood. Different letters indicate significant difference at *P* < 0.05.

To confirm the results shown earlier, we conducted syringe assays at 14°C. Survival of males in a 10-ml syringe compacted down to 1 ml (1 cm^3^) stayed relatively consistent (over 70%) regardless of the number of males placed in the volume (Fig. 3). There was a steep drop off in survival from 30 to 40 mosquitoes (approximately 94% survival to 75% survival), with a gradual increase from 50 to 80 mosquitoes and then another drop from 80 to 160 mosquitoes (93.9% down to 79.6%), and back to an increased survival rate of 90.7% at 240 mosquitoes compacted at 1 ml of volume. It should be noted that there was only one compaction rate that was statistically different from the rest and that was at 40 mosquitoes per 1 ml of volume (*P* < 0.05).

### High Compaction Rates Result in Higher Individual Damage and Lesser Mortality During Transport

**Fig. 4.**
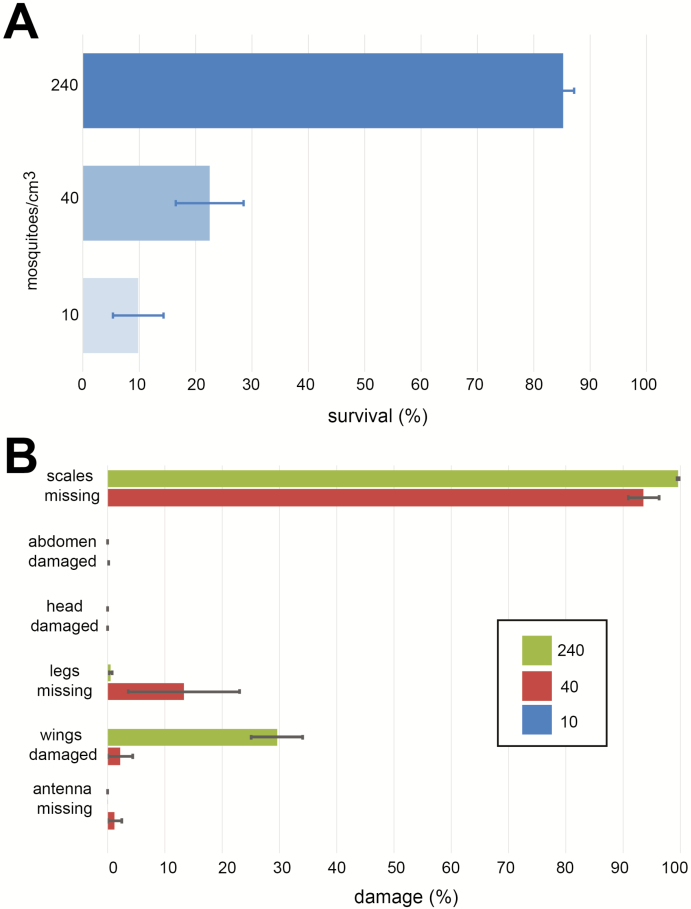
Survival and damage after overnight shipping of mosquitoes from Las Cruces, NM, to Davis, CA. Averages of at least five biological replicates are shown; error bars represent SE. (A) The survival percentages for different compaction rates. (B) Percent damage at specific structures for males that survived the overnight shipping by compaction rates.

The time from packaging male mosquitoes in New Mexico to their release into a cage in California was approximately 20 h. The data from the temperature tracker showed that the temperature in the package fluctuated between 7 and 14°C during shipping. Fig. 4A shows survival rates of *Ae. aegypti* mosquito males after overnight shipping from New Mexico to California. We recorded survival rates of 9.8% for mosquito males that were shipped at 10 individuals/cm^3^, 22.5% for males that were shipped at 40 individuals/cm^3^, and 85.2% for males that were shipped at 240 individuals/cm^3^. A high number of mosquitoes that were shipped at the 240 and 40 individuals/cm^3^ compaction rate had missing scales (98 and 94%, respectively), whereas none of the survivors at the lowest compaction rate were missing scales (Fig. 4B). Of the highly compacted males, 29.6% showed some wing damage primarily on the fringes of the wings. Despite the wing damage, all males demonstrated an ability to fly within the BugDorm cages. Less than 1% of surviving mosquitoes in the highly compacted groups were missing legs. Other structures we examined in surviving mosquitoes, abdomens, heads, and antennae did not show any significant damage in any compaction group.

## Discussion

Mosquito SIT has great potential to become an important part of the vector control ‘toolbox’ in the future. Its potential is especially high when used in combination with other interventions as part of an integrated pest management strategy. In our opinion, SIT may become an invaluable tool for endgame scenarios when vector populations are extremely low due to other interventions such as insecticide spraying (Alphey et al. 2010). Under such conditions, sterile males will be much more effective in finding and neutralizing remaining female mosquitoes than a human pest control agent. To be successful, mosquito SIT has to be cost effective. Streamlined protocols for the production and sterilization of mosquito males are as important as transport protocols that ensure maximum survival and minimum damage to mosquito males during transport from the rearing facility to the release sites.

Mosquito SIT has been successfully tested in several small-scale studies to reduce populations of *Ae. aegypti* (Lacroix et al. 2012, Carvalho et al. 2015) and *Aedes albopictus* Skuse (Diptera: Culicidae) (Bellini et al. 2007). The fact that *Ae. aegypti* males have relatively short dispersal ranges compared with other insects complicates implementation of large-scale SIT programs (Reiter et al. 1995, Harrington et al. 2005, Alphey et al. 2010, Carvalho et al. 2015). We propose that instead of centralized ground releases of large number of males, aerial releases of smaller groups of males via unmanned aerial vehicles (UAVs) will enable us to better cover a given intervention area. The development and deployment of UAS for use in SIT applications related to pink bollworm (*Pectinophora gossypiella* Saunders [Lepidoptera: Gelechiidae]); Moses-Gonzales and Walters 2015) and codling moth (*Cydia pomonella* Linnaeus [Lepidoptera: Tortricidae]) demonstrate the efficacy of a single UAS deployed to release sterile insects. In larger-scale pest programs deploying SIT as a component of AW-IPM, the use of swarm technology is currently under development (Moses-Gonzales 2017). During March 2018, FAO/IAEA released sterile *Ae. aegypti* from UAS in Brazil. The preliminary results are still undergoing analysis. Several drone-release systems for mosquitoes are currently in development. These devices range in size, scale, and complexity. Several design strategies are being explored; however, it is critical to address biological concerns, especially in terms of handling, transport, and release, prior to committing to a single design.

In most realistic scenarios, the rearing/sterilization sites and release sites of SIT insects are located at considerable distances. Previously, packing for transportation of the screwworm fly has included low-temperature immobilization (Smittle and Patterson 1974); carbon dioxide, nitrogen, and cold combinations (Hooper 1970); low-oxygen pressure chambers (Tanaka et al. 1972); or simply under ambient conditions (Snow et al. 1971, Chambers 1977).

The results of our incubator studies (Fig. 1) suggest that *Ae. aegypti* males can tolerate a wide range of storage temperatures for the first 24 h. Mortality at the 48-h time point and all later time points was significantly increased at all temperatures we tested. We therefore recommend to avoid transport and storage times longer than 24 h for male mosquitoes.

The results of the compaction studies that we performed in the laboratory environment using the larger 10 cm^3^ chambers suggested that a compaction rate of 40 mosquitoes/cm^3^ results in better survival than the higher compaction rate of 80 mosquitoes/cm^3^ (Fig. 2). Surprisingly, the effects of different levels of compaction were quite different when we performed similar incubator experiments using syringes with 1 cm^3^ chambers. In this setup, a compaction rate of 40 mosquitoes/cm^3^ produced significantly lower survival rates than all other compaction rates we tested (Fig. 3). Even compaction to 240 mosquitoes/cm^3^, the highest compaction rate we could achieve without squeezing the males to death did not result in significant mortality. We hypothesize that a density of 40 mosquitoes/cm^3^ leads to a higher number of damaging interactions between individual males than happen at the other compaction rates. Additional studies are necessary to test this hypothesis.

Based on our findings, we decided to perform a real-life shipping assay using syringes as transport chambers and determine survival as well as damage to the mosquito males that were shipped at different compaction rates. The results of the shipping assay clearly show that the highest compaction rate we tested (240 males/cm^3^) resulted in the highest survival rates (Fig. 4A). Although a high percentage of mosquito males that were compacted with this rate were missing scales, we did not find a single broken antenna in the debris we analyzed. A high percentage of the highly compacted group showed some wing damage, but this damage was mainly at the fringes of the wings and these males were still able to fly. A likely explanation for the improved survival at higher compaction rates is that the highly compacted males are less damaged by the vibrations during transport by airplane and vehicle. Further experiments are needed to test this hypothesis. Further experiments are also needed to assess the impact of transport damage on male survival, fitness, and mating success. Also, ‘real-life’ drone-release experiments will have to be performed to test the effect of transport and storage conditions on these parameters.

In summary, our results show that lower compaction rates produce less individual damage but significantly higher mortality rates in our real-life overnight shipping assay. Mosquito survival at the highest compaction rate we tested was 85%. This is good news because many professional courier delivery services offer ‘overnight shipping’ within the United States, thereby providing a suitable transport chain for sterile male mosquitoes from rearing facilities to their release sites.

## Supplementary Material

Supplemental File
